# Evaluation of a home pharmaceutical care service model for home-based patients receiving anticoagulation therapy within county-level medical community

**DOI:** 10.1371/journal.pone.0339834

**Published:** 2026-01-05

**Authors:** JianAo Song, Bin Chen, Jinhui Sun, KaiZhan Zhuang

**Affiliations:** 1 Department of Pharmacy, the People’s Hospital of Fenghua Ningbo, Ningbo, Zhejiang, China; 2 Medical Management Center, the People’s Hospital of Fenghua Ningbo, Ningbo, Zhejiang, China; Siloam Hospitals Lippo Village, INDONESIA

## Abstract

**Objective:**

To establish a systematic, standardized, and operational model of home pharmaceutical care for patients with anticoagulant treatment in county-level medical community.

**Methods:**

A home pharmaceutical care service team was constituted by pharmacists and physicians from the county-level medical community. A home-based pharmaceutical care survey was conducted for patients with long-term oral anticoagulants (VKAs, rivaroxaban, dabigatran and apixaban). The patients’ awareness and adherence score to oral anticoagulants(OATs) was evaluated before and after home pharmaceutical care service. The awareness and adherence score were evaluated with awareness questionnaires and Morisky Medication Adherence Scale-8 (MMAS-8), respectively. The paired-sample t tests were used to compare the awareness and adherence score.

**Results:**

Finally, totally 95 patients completed the survey. Among these patients, 29 patients were in the VKAs group and 66 patients were in the NOACs group. Following the implementation of home pharmaceutical care service, there was an increase in the awareness score, rising from 5.02 ± 1.71 to 8.09 ± 1.25 (*p* < 0.001). Similarly, the adherence score increased from 4.66 ± 1.66 to 6.94 ± 0.59 (*p* < 0.001).

**Conclusions:**

This pilot study demonstrates that the home pharmaceutical care service model implemented within county-level medical community for anticoagulation treatment is feasible and shows promise in improving anticoagulation knowledge and medication adherence among patients.The findings provide preliminary evidence for a service model that could be further evaluated. Future controlled studies are warranted to confirm its efficacy and to investigate its impact on clinical health outcomes.

## Introduction

Oral anticoagulants (OAT) are efficacious in preventing the formation of blood clots and are extensively utilized in the treatment and prevention of various thromboembolic and cardiovascular diseases [1–3]. Currently, the most commonly used oral anticoagulants in clinical practice include oral vitamin-K antagonists (VKA, warfarin) and non-vitamin K antagonists (NOACs, including dabigatran, rivaroxaban and apixaban) [4–6]. VKA and NOACs are classified as high-alert medications in numerous countries due to their inherent pharmacodynamic properties. Studies indicated that certain conditions, such as inappropriate prescription, underdosed, inadequately stored, non-adherence to medical advice, all of these will increase the risk of drug-related complications, including hemorrhagic or thrombotic events [2,7]. Data from the US National Electronic Injury Surveillance System-Cooperative Adverse Drug Event Surveillance (NEISS-CADES) revealed that over the five-year period from 2016 to 2020, there were 5.9 emergency department (ED) visits per 100 patients dispensed NOACs while 13.0 ED visits per 100 patients dispensed warfarin, for oral anticoagulant-related bleeding [8]. Meanwhile, the prescriptions of OAT have increased rapidly worldwide due to the increasing older population. Therefore, it is essential to enhance the management of anticoagulation therapy.

The European Heart Rhythm Association (EHRA) and the European Society of Cardiology (ESC) guidelines indicated that follow-up of OAT-patients is essential for patient safety as various health care providers are involved in providing anticoagulation care, including physicians, nurses and pharmacists [3,9,10]. The participation of pharmacists in the management of anticoagulant drugs is very beneficial for patients, which could reduce the incidence of adverse drug events and hospital readmissions. To date, few programs in the literature have described the home pharmaceutical care model covering VKA and NOACs. For anticoagulation patients who have completed hospitalization and outpatient treatment, most of the time they take medication at home. However, the traditional pharmaceutical care model is limited to hospitalization and outpatient services, which cannot meet the patients’ need for continuous medical care. Thus, home pharmaceutical care, which could deliver individualized, whole-process and continuous pharmaceutical services and health education is an appropriate choice. Home pharmaceutical care could play an effective role in home management of anticoagulation, which may be a critical aspect of ensuring patient safety and treatment efficacy [11,12]. Consequently, we aim to promote and develop a home pharmaceutical care service model for home patients with oral anticoagulants treatment including both VKA and NOACs in county-level medical community in China.

## Materials and methods

### Study design and patients’ population

The study was performed on patients attending clinic at the county-level medical community hospital in Ningbo, China between November 2022 and November 2023. The inclusion criteria were as follows: (1) patients receive OAT therapy (including warfarin, rivaroxaban, apixaban, and dabigatran) at home; (2) patients should be conscious and could complete the questionnaire independently; (3) patients should be voluntary, and sign the written informed consent. The exclusion criteria were as follow: (1) patients were diagnosed with cognitive impairments or active psychiatric conditions, (2) patients were unable to participate due to physical reasons (e.g., requiring hospitalization for unstable clinical status). The termination criteria included failure to follow up for various reasons and death. This study adopted an exploratory pilot design without formal sample size calculation, so the statistical power was limited. The sample size was determined by clinical feasibility and real-world availability of eligible participants during the study period. This study was approved by the ethics committee of the People’s Hospital of Fenghua Ningbo (Ethical number:2022–29-K).

### Home pharmaceutical care service

The service team was constituted of integrating seven pharmacists from the county-level medical community, alongside three physicians specializing in cardiology, respiratory medicine and neurology, respectively. Prior to initiating the survey, all team members undergo rigorous standardized training. The pharmacists will visit patients to gather the basic information of patients and help patients have a comprehensive understanding of the services they can receive. It is crucial that pharmacists need to confirm that the patient has complete understanding of the service and obtain their voluntary signed written informed consent voluntarily. Subsequently, pharmacists will conduct an analytical evaluation of patients’ awareness and adherence to OAT by guiding patients to complete the relevant questionnaires, thereby obtaining the essential baseline data.

**Fig 1:**
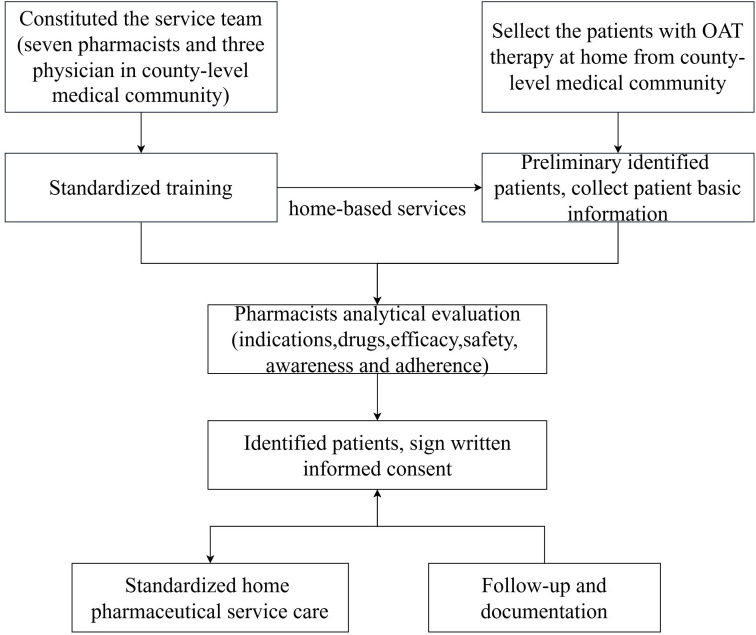
The home pharmaceutical care service process.

In the next period, the service team will deliver standardized home pharmaceutical service care to patients, including medical record review, medication reconciliation, interpreting laboratory results (e.g., international normalized ratio, INR), providing patient with education and counseling, and promptly reporting medication errors and adverse drug reactions. Follow-up assessments will be conducted at three and six months later. If the patient cannot be reached at these time points, they will be considered as “lost to follow up”. The specific process of home pharmaceutical care service process is shown in Fig 1.

### Patient awareness and adherence assessment

Patients’ awareness of OAT was assessed using an awareness questionnaire developed through discussion among the service team members. The awareness questionnaire was designed by drawing on published articles [13,14]. Its content validity was then established through rigorous evaluation by a multidisciplinary expert panel (7 clinical pharmacists, 3 physicians). Based on the feedback from the expert group, the expression and content of the questionnaire were modified and improved, resulting in the final version. The final version awareness questionnaire containing 10 items, which all are yes/no questions, and the specific items are shown in S1 Table. The total score ranges from 0 to 10, with higher scores representing higher awareness levels.This process ensures that the questionnaire can comprehensively and accurately measure the level of awareness of anticoagulant therapy patients.

Patient adherence to OAT was assessed by the Morisky Medication Adherence Scale-8 (MMAS-8), which is one of the most frequently used patient questionnaires for the assessment of medication adherence [15]. The MMAS-8 consists of 8 items, the first 7 of which are yes/no questions, and the last of which is a 5-point Likert-scale rating (S2 Table). Scores are summed and range from 0–8, the higher score indicating the higher patients’ adherence level. To minimize potential language barriers and ensure optimal comprehension, both the awareness questionnaire and MMAS-8 were conducted in validated Chinese versions. The Chinese version of the MMAS-8 has shown good internal consistency in Chinese patient populations, as evidenced by Cronbach’s α values of 0.74 and 0.77 in studies of chronic cardiovascular disease and myocardial infarction patients, respectively [16,17]. Meanwhile, all assessments were conducted following the standardized protocols to maintain methodological consistency.

### Statistical analysis

The MMAS-8 and awareness questionnaire were measured before and after the home pharmaceutical care service. All data analyses were conducted with IBM SPSS software (version 26.0). Continuous variables were described in the form of mean and standard deviations (SD). The scores were compared using paired-sample t tests. The significance level was set at *p *< 0.05.

## Results

### Patients characteristics

**Fig 2:**
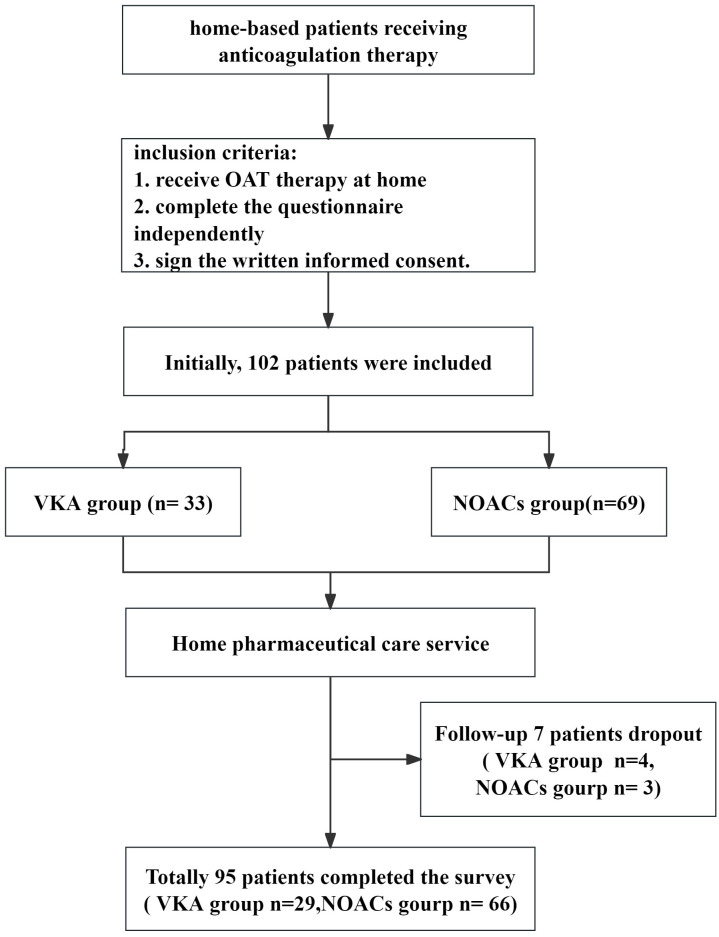
The detailed participant flow diagram.

A total of 102 patients participated in the survey on home pharmaceutical care service. During the follow-up period, 7 patients (6.86%) were lost to follow up. Detailed reasons for dropout were not systematically recorded, through it is noted that some participants discontinued their involvement was related to the burden of home-visit services (Fig 2). Ultimately, 95 patients completed the survey. The baseline characteristics of 95 completers vs 7 dropouts were compared, which incicated that there was no attrition bias (S3 Table). Among these patients, 29 were receiving VKA therapy, while the remaining 66 were on NOACs therapy. For the NOACs therapy 49 received rivaroxaban, 15 dabigatran, and 2 apixaban. The average age of all the participant patients involved in the study was 67.5 ± 8.0, and 61 (64.21%) were female. For the 95 patients, majority of patients (65,68.42%) were aged ≥ 65 years. The main indications for all OAT patients were atrial fibrillation (63,66.31%), mechanical valve (20,21.05%), and thromboembolism (12,12.63%). The OAT duration of a large proportion of patients was between 1 and 5 years (56,58.94%). In these patients, hypertension was the most common comorbidity (47, 49.47%), followed by diabetes mellitus (21,22.11%). Only 27 (28.42%) patients didn’t receive the combined therapy. Approximately 36 patients (40.9%) reported an educational background of senior high school or higher, while 59 patients (59.6%) held a junior high school or lower. The urban-rural distribution was relatively balanced, with 50 patients (50.5%) residing in urban areas and 45 (45.5%) in rural regions. The baseline characteristics of participant patients are shown in Table 1.

**Table 1:** Basic characteristics of patients (n = 95).

Variable	VKA(n = 29)	NOACs (n = 66)	Total OAT (n = 95)
**Age, mean**±**SD**	58.3 ± 11.8	71.5 ± 10.9	67.5 ± 8.0
**Age group**			
< 65	17	14	31
≥ 65	12	52	64
**Sex**			
Male	15	46	34
Female	14	20	61
**Indication**			
Atrial Fibrillation	7	56	63
Mechanical Valve	20	0	20
Thromboembolism	2	10	12
**Comorbidities**			
Hypertension	9	38	47
Diabetes mellitus	6	15	21
Other chronic diseases	4	12	16
**OAT Duration**			
< 1 years	6	25	31
1 ~ 5 years	17	39	56
> 5 years	6	2	8
**Combination therapy**			
No	10	17	27
Yes	19	49	68
**Educational background**			
Senior high school or above	9	27	36
Junior high school or below	12	47	59
**Distribution**			
Urban	17	33	50
Rural	22	23	45

### Patient awareness and adherence evaluation

**Fig 3:**
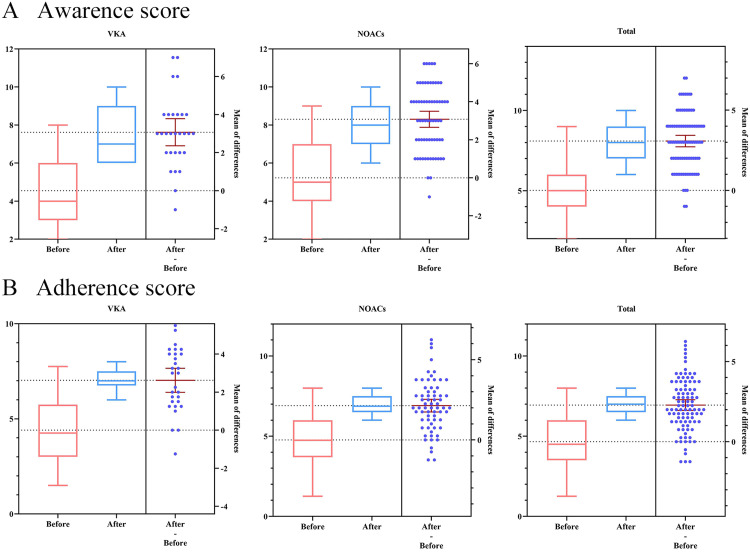
Patient awareness and adherence evaluation. (A) The awareness score of home-based patients undergoing anticoagulation treatment before and after receiving home pharmaceutical care service. (B) The adherence score of home-based patients undergoing anticoagulation treatment before and after receiving home pharmaceutical care service.

Meanwhile, the awareness of OAT was evaluated by an awareness questionnaire. Detailed results were presented in Fig 3A and Table 2. Initially the awareness score of all patients to OAT was 5.02 ± 1.71. Following the provision of home pharmaceutical care service, this score significantly increased to 8.09 ± 1.25 (*p* < 0.001). Specifically, in the VAK group, the awareness score increased from 4.55 ± 1.55 to 7.62 ± 1.40 (*p* < 0.001), while in the NOACs group it increased from 4.77 ± 1.65 to 6.91 ± 0.61 (*p* < 0.001).

**Table 2:** Adherence and awareness score in patients taking OAT comparison before and after Home pharmaceutical care service.

Group	N	Before	After	*p* Value
**Adherence score**				
VKA	29	4.41 ± 1.69	7.02 ± 0.55	<0.001
NOACs	66	4.77 ± 1.65	6.91 ± 0.61	<0.001
Total	95	4.66 ± 1.66	6.94 ± 0.59	<0.001
**Awareness score**				
VKA	29	4.55 ± 1.55	7.62 ± 1.40	<0.001
NOACs	66	5.23 ± 1.74	8.30 ± 1.14	<0.001
Total	95	5.02 ± 1.71	8.09 ± 1.25	<0.001

The adherence of patients to the OAT was assessed using the MMAS-8. As illustrated in Fig 3B and Table 2, there was a statistically significant increase in the patients’ OAT adherence score, from 4.66 ± 1.66 to 6.94 ± 0.59 (*p* < 0.001), after receiving comprehensive home pharmaceutical care service by our service team. Specifically, the adherence score increased from 4.41 ± 1.69 to 7.02 ± 0.55 in VAK group (*p* < 0.001), and from 4.77 ± 1.65 to 6.91 ± 0.61 in NOACs group (*p* < 0.001), respectively.

### Other clinical outcomes

During the entire home pharmaceutical care service process, our team assisted a total of 95 individuals with medication reconciliation and helped 32 patients avoid inappropriate medication use. Among the 29 patients treated with warfarin, 28 maintained their INR within the therapeutic target range. Additionally, during the trial period, 4 patients suffered thromboembolic episodes, 3 of which were in the NOACs group. Furthermore, 3 bleeding events were recorded, and 2 patients hospitalized. The detail information were displayed in Table 3.

**Table 3:** Other clinical outcomes during home pharmaceutical care service.

	VKA (n = 29)	NOACs (n = 66)	Total OAT (n = 95)
Medication Reconciliation	29	66	95
Avoiding inappropriate medication use	11	21	32
INR stability	28	–	28
Thromboembolic episodes	1	3	4
Bleeding events	2	1	3
Hospitalizations	2	0	2

## Discussion

Oral anticoagulation therapy (OAT) is one of the most common pharmacological interventions for patients with cardiovascular diseases and thromboembolic diseases. The management of OAT increasingly emphasizes home-based medication monitoring and administration. The management of home patients with anticoagulation treatment at home is a critical aspect of ensuring patient safety and treatment efficacy. The home-based management of anticoagulation has garnered attention due to its potential to improve patient adherence and enhance overall treatment outcomes [18]. Various studies have highlighted the importance of medication adherence in managing chronic conditions, as non-adherence can lead to worsening health outcomes [19,20]. However, approximately half of the patients will face challenges in self-managing their anticoagulation therapy due to various issues such as cognitive abilities and educational background. Therefore, ongoing education for both healthcare providers and patients is especially vital for the management of OAT therapy in the home setting [21]. We develop a home pharmaceutical care service model in county-level medical community, which could significantly increase the adherence and awareness of home patients to OAT. The results demonstrated that the home pharmaceutical care service model is an effective way to enhance the home OAT patients management, which could help to improve the safety and effectiveness of OAT.

Patients’ knowledge and understanding generally has a direct impact on the benefit of the drug. Assessing and improving patients’ OAT awareness can lead to better treatment outcomes. We administered a comprehensive survey to patients, which included various dimensions such as the rationale for medication use, proper administration techniques, risks associated with discontinuation, and the management of adverse reactions. Based on the analysis of the survey data, we developed targeted medication service plans that prioritized educational interventions in identified areas of deficiency. This initiative aimed to effectively enhance patients’ understanding of anticoagulant medications. Notably, the score of patients’ OAT awareness increased with the home pharmaceutical care service. This outcome indicated that our home pharmaceutical care service model for home patients with anticoagulation treatment in county-level medical community is viable and effective.

The Morisky medication adherence scale as an indirect assessment of adherence, which refers to the active involvement of patients in their treatment. Improving patient adherence is essential for proper treatment. Prior studies emphasized that in patients using OAT, adherence to and persistence with OAT therapy should be assessed and supported [22]. Consequently, improving patient adherence has become a critical priority. In our survey, the mean adherence score for all patients was 4.66, which indicated poor adherence. The reasons for such poor adherence may be as follows: lack of comprehensive understanding of medication, concerns about drug side effects, interminable treatment, unawareness of the hazards of not taking medication on time, advanced age, and other factors. A study showed that the better medication knowledge, female sex, and no history of venous thromboembolism were associated with better adherence in Vietnam OAT patients [23]. At the conclusion of the survey, the adherence score increased to 6.94 ± 0.59. Meanwhile, the majority of patients transitioned from low adherence to medium adherence, with some achieving high adherence. With the implementation of home pharmaceutical care services, 92.63% of patients showed medium adherence (S4 Table). All of these findings suggested that the home pharmaceutical care service model is viable within the county-level medical community to assist home-based patients undergoing OAT. Furthermore, no significant difference in adherence score was observed between patients taking VKA and those taking NOACs, which was consistent with other published studies which showed that similar adherence was noted between NOACs and VKA regardless of the frequency of serum level monitoring [22].

This study was carried out in a county-level medical community hospital in Ningbo, China. It should be noted that the patient population, local healthcare practices, and available resources may differ from hospitals in other cities. Therefore, the generalizability of our findings to wider populations may be limited. Additionally, The home pharmaceutical care service described in this study involved notable resource needs, including costs for training, transportation, and dedicated follow-up time, which must be addressed for successful scaling. Future research should involve larger, multicenter trials to verity the intervention’s efficacy and assess its replication and broader applicability.

Several limitations of this study should be acknowledged. First, the single-arm pre-post design without a control group, limits our ability to draw causal inferences regarding the intervention’s effects. Although significant improvements in outcomes was observed, the confounding factors cannot be excluded. Second, the findings of this pilot study should be interpreted in the context of its statistical approach. Only paired t-tests were used in our analysis, without adjustment for potential confounders such as age, educational background and comorbidities. These unadjusted variables may have influenced the observed effect size. For instance, participants with higher education levels might have benefited more from the educational components of the intervention. Therefore, the results of our study should be interpreted with caution, and future studies should prioritize a controlled design or the use of multivariate analytical techniques to isolate the true effect of the intervention from other patient characteristics. Third, the single-center design and sample size may limit the generalizability of the findings. While the study successfully established a feasible service model within a county-level medical community and provided preliminary effect estimates, the results may not be fully transferable to other settings or patient populations. In addition, the limited sample size restricted deeper analysis of the relationships between patient characteristics and adherence outcomes.Fourth, methodological constraints may have introduced potential bias. The same team was responsible for both delivering the intervention and collecting outcome data, which could lead to interviewer bias or Hawthorne effects. Future studies would benefit from using independent assessors to minimize these risks. Finally, the primary outcomes for this study are awareness and adherence scores, which limited the assessment of harder clinical endpoints (e.g., bleeding, INR stability, hospitalization), although we simply record the some clinical outcomes. Longer-term studies with more than 12-month follow-up tracking both adherence metrics and clinical endpoints are needed to validate the real-world impact of this home pharmaceutical care service model.

Despite these limitations, this study offers a valuable proof-of-concept for implementing home-based pharmaceutical care within county-level medical communities. Future research should focus on conducting controlled, multicenter trials with larger samples, and longer follow-up. Further investigation is also needed to identify factors influencing adherence and to optimize strategies for sustaining patient engagement and improving clinical outcomes in real-world practice.

## Conclusion

The awareness and adherence of patients play a crucial role in optimizing the benefit of OAT therapy, potentially enhancing the treatment safety and clinical outcomes. This pilot study demonstrated that the home pharmaceutical care service model implemented within a country-level medical community is feasible and shows promise in improving anticoagulation knowledge and medication adherence among home-based patients (p < 0.05). By integrating pharmaceutical care into patients’ daily life, this model effectively addressed the specific needs of home-based patients undergoing anticoagulation therapy. Additionally, it could facilitate the personalized medication management, monitor the therapeutic effects continuously, and provide education to targeted patients, all of which contribute to improved awareness and adherence. This study provides preliminary experience for facilitate future multicenter investigations and broader population studies, and offer an empirical foundation for developing evidence-based strategies to optimize home-based anticoagulant management protocols.

## Supporting information

S1 Table The awareness questionnaire for Oral anticoagulants (OAT). (DOCX)

S2 Table The 8-item Morisky Medication Adherence Scale (MMAS-8). (DOCX)

S3 Table Baseline characteristics of Complete patients(n = 95), dropouts patients(n = 7) and all patients(n = 102). (DOCX)

S4 Table Adherence Rating of Patients Taking OAT. (DOCX)
