# Supplementary material for: Evaluation of a home pharmaceutical care service model for home-based patients receiving anticoagulation therapy within county-level medical community
Source: PLoS One. 2026 Jan 5;21(1):e0339834. doi: 10.1371/journal.pone.0339834 (PMC12768357; doi:10.1371/journal.pone.0339834)
Supplement: S1 Table — (DOCX) [file pone.0339834.s001.docx]

**S1 Table : The awareness questionnaire for Oral anticoagulants(OAT).**

| **Awareness questions** | Patients Response |
| --- | --- |
| 1. Do you know which anticoagulant medication you are taking? | Yes[1]  No[0] |
| 1. Do you know the reason why you are taking anticoagulant medication? | Yes[1]  No[0] |
| 1. Do you know the dosage and frequency of the OAT medication you are currently taking every day? | Yes[1]  No[0] |
| 1. Do you know the common side effects of taking oral anticoagulant medications? | Yes[1]  No[0] |
| 1. Do you know what to do if you experience any adverse reactions? | Yes[1]  No[0] |
| 1. Do you understand the potential risks of not following your doctor's instructions for medication use? | Yes[1]  No[0] |
| 1. Do you know what to do if you forget to take your medication on time or miss a dose? | Yes[1]  No[0] |
| 1. Do you know what precautions you need to take when taking anticoagulants? | Yes[1]  No[0] |
| 1. Do you know the blood tests or kidney function needsare necessary? | Yes[1]  No[0] |
| 1. Do you know that if you need to undergo invasive surgical treatment, you should inform your doctor that you are currently taking anticoagulants? | Yes[1]  No[0] |
