# Supplementary material for: Evaluation of a home pharmaceutical care service model for home-based patients receiving anticoagulation therapy within county-level medical community
Source: PLoS One. 2026 Jan 5;21(1):e0339834. doi: 10.1371/journal.pone.0339834 (PMC12768357; doi:10.1371/journal.pone.0339834)
Supplement: S2 Table — (DOCX) [file pone.0339834.s002.docx]

**S2 Table : The 8-item Morisky Medication Adherence Scale (MMAS-8).**

| **MMAS-8 adherence questions** | **Patients Response** |
| --- | --- |
| 1.Do you ever forget to take your medicine? | Yes[0]  No[1] |
| 2.Over the past 2 weeks, were there any days when you did not take your high blood pressure medicine? | Yes[0]  No[1] |
| 3.Have you ever cut back or stopped taking your medication without telling your doctor because you felt worse when you took it? | Yes[0]  No[1] |
| 4.When you travel or leave home, do you sometimes forget to bring along your medications? | Yes[0]  No[1] |
| 5.Did you take your high blood pressure medicine yesterday? | Yes[0]  No[1] |
| 6.When you feel like your blood pressure is under control, do you sometimes stop taking your medicine? | Yes[0]  No[1] |
| 7.Taking medication everyday is a real inconvenience for some people. Do you ever feel hassled about sticking to your blood pressure treatment plan? | Yes[0]  No[1] |
| 8.How often do you have difficulty remembering to take all your blood pressure medication? | Never[1]  Once in a while[0.75]  Sometimes[0.5]  Usually[0.25]  all the time[0] |
