# Supplementary material for: Evaluation of a home pharmaceutical care service model for home-based patients receiving anticoagulation therapy within county-level medical community
Source: PLoS One. 2026 Jan 5;21(1):e0339834. doi: 10.1371/journal.pone.0339834 (PMC12768357; doi:10.1371/journal.pone.0339834)
Supplement: S3 Table — (DOCX) [file pone.0339834.s003.docx]

**S3 Table: Baseline characteristics of Complete patients(n=95) ,dropouts patients(n=7) and all patients(n=102).**

|  | **Completers(n=95)** | **dropouts(n=7)** | ***p*** | **Completers(n=95)** | **All patients(n=102)** | ***p*** |
| --- | --- | --- | --- | --- | --- | --- |
| Age,mean±SD | 67.5±12.7 | 69.57±12.46 | 0.647 | 67.5±12.7 | 67.62±12.64 | 0.937 |
| Age group |  |  | 0.89 |  |  | 0.917 |
| < 65 | 31 | 3 |  | 31 | 34 |  |
| ≥ 65 | 64 | 4 |  | 64 | 68 |  |
| Sex |  |  | 1.00 |  |  | 0.942 |
| Male | 34 | 2 |  | 34 | 36 |  |
| Female | 61 | 5 |  | 61 | 66 |  |
| Indication |  |  | 0.179 |  |  | 0.942 |
| Atrial Fibrillation | 63 | 7 |  | 63 | 70 |  |
| Mechanical Valve | 20 | 0 |  | 20 | 20 |  |
| Thromboembolism | 12 | 0 |  | 12 | 12 |  |
| Comorbidities |  |  | / |  |  | / |
| Hypertension | 47 | 0 |  | 47 | 47 |  |
| Diabetes mellitus | 21 | 0 |  | 21 | 21 |  |
| Other chronic diseases | 16 | 4 |  | 16 | 20 |  |
| OAT Duration |  |  | 0.22 |  |  | 0.944 |
| < 1 years | 31 | 2 |  | 31 | 33 |  |
| 1~5 years | 56 | 3 |  | 56 | 59 |  |
| > 5 years | 8 | 2 |  | 8 | 10 |  |
| Combination therapy |  |  | 0.711 |  |  | 0.879 |
| No | 27 | 1 |  | 27 | 28 |  |
| Yes | 68 | 6 |  | 68 | 74 |  |
