# Supplementary material for: Evaluation of a home pharmaceutical care service model for home-based patients receiving anticoagulation therapy within county-level medical community
Source: PLoS One. 2026 Jan 5;21(1):e0339834. doi: 10.1371/journal.pone.0339834 (PMC12768357; doi:10.1371/journal.pone.0339834)
Supplement: S4 Table — (DOCX) [file pone.0339834.s004.docx]

**S4 Table: Adherence Rating of Patients Taking OAT.**

| **Adherence Rating** | **VKA(n=29)** | | **NOAC(n=66)** | | **Total(n=95)** | |
| --- | --- | --- | --- | --- | --- | --- |
|  | **Before** | **After** | **Before** | **After** | **Before** | **After** |
| **Low adherence** | 23 | 0 | 47 | 0 | 70 | 0 |
| **Medium adherence** | 6 | 26 | 17 | 62 | 23 | 88 |
| **High adherence** | 0 | 3 | 2 | 4 | 2 | 7 |

Patients are considered to have low adherence with scores less than 6, medium adherence with scores of 6 to 7, and high adherence with a score of 8.
